# Is the Oxidant/Antioxidant Status Altered in CADASIL Patients?

**DOI:** 10.1371/journal.pone.0067077

**Published:** 2013-06-14

**Authors:** Jonica Campolo, Renata De Maria, Caterina Mariotti, Chiara Tomasello, Marina Parolini, Marina Frontali, Domenico Inzitari, Raffaella Valenti, Antonio Federico, Franco Taroni, Oberdan Parodi

**Affiliations:** 1 Community Networking Resources Institute of Clinical Physiology, Cardiothoracic and Vascular Department, Niguarda Ca' Granda Hospital, Milan, Italy; 2 Unit of Genetics of Neurodegenerative and Metabolic Diseases, Fondazione IRCCS Istituto Neurologico Carlo Besta, Milan, Italy; 3 Community Networking Resources Institute of Translational Pharmacology, Rome, Italy; 4 Department of Neurological and Psychiatric Sciences, University of Florence, Florence, Italy; 5 Department of Neurological, Neurosurgical and Behavioural Sciences, University of Siena, Siena, Italy; Center for Cancer Research, National Cancer Institute, United States of America

## Abstract

The altered aggregation of proteins in non-native conformation is associated with endoplasmic reticulum derangements, mitochondrial dysfunction and excessive production of reactive oxygen species. Cerebral autosomal dominant arteriopathy with subcortical infarcts and leukoencephalopathy (CADASIL) is a rare hereditary systemic vasculopathy, caused by *NOTCH3* mutations within the receptor extracellular domain, that lead to abnormal accumulation of the mutated protein in the vascular wall. *NOTCH3* misfolding could cause free radicals increase also in CADASIL. Aim of the study was to verify whether CADASIL patients have increased oxidative stress compared to unrelated healthy controls. We enrolled 15 CADASIL patients and 16 gender- and age-matched healthy controls with comparable cardiovascular risk factor. Blood and plasma reduced and total aminothiols (homocysteine, cysteine, glutathione, cysteinylglycine) were measured by HPLC and plasma 3-nitrotyrosine by ELISA. Only plasma reduced cysteine (Pr-Cys) and blood reduced glutathione (Br-GSH) concentrations differed between groups: in CADASIL patients Br-GSH levels were higher (p = 0.019) and Pr-Cys lower (p = 0.010) than in controls. No correlation was found between Br-GSH and Pr-Cys either in CADASIL patients (rho 0.25, *P*=0.36) or in controls (rho -0.15, *P*=0.44). Conversely, 3-nitrotyrosine values were similar in CADASIL and healthy subjects (p = 0.82). The high levels of antioxidant molecules and low levels of oxidant mediators found in our CADASIL population might either be expression of an effective protective action against free radical formation at an early stage of clinical symptoms or they could suggest that oxidative stress is not directly involved in the pathogenesis of CADASIL.

## Introduction

Perturbation of the oxidant/antioxidant balance within the cells has been suggested to be involved in the pathogenesis of several neurodegenerative disorders such as stroke [1], Parkinson’s disease [2] and Alzheimer’s disease [3]. These neurological disorders are associated with the production of abnormally aggregated proteins and belong to the group of protein conformational diseases.

The altered aggregation of proteins in non-native conformation is generally associated with derangements of the endoplasmic reticulum and stress, which leads to mitochondrial dysfunction and excessive production of reactive oxygen species (ROS) [4]. When the rate of free radical generation exceeds the capacity of antioxidant defenses, oxidative stress ensues causing extensive damage to DNA, proteins and lipids.

An increasing body of evidence suggests that dysfunction of cell energy metabolism is an important factor in neurotoxicity mediated by nitric oxide (NO) and that the intracellular content of thiols is crucial in determining the sensitivity of cells to oxidative and nitrosative stress [5]. The actions of NO can be either direct, resulting from reactions between NO and specific biological molecules, or indirect, resulting from reactions of NO-derived reactive nitrogen species. For instance, the reaction of NO with superoxide produces the peroxynitrite anion and represents an important pathway of NO reactivity [6]. Peroxynitrite is a powerful oxidant and can nitrate aromatic amino acid residues such as tyrosine to form nitrotyrosine.

Cerebral autosomal dominant arteriopathy with subcortical infarcts and leukoencephalopathy (CADASIL) is a rare hereditary systemic vasculopathy, caused by mutations in the *NOTCH3* gene encoding a transmembrane receptor mainly expressed in vascular smooth muscle cells (VSMC) in adult human tissue [7]. The notion of CADASIL as a systemic disease is supported by the finding of diffuse structural small vessel abnormalities not only in the brain, but also in the skin, nerves, and muscles [7]. Furthermore altered endothelium-dependent vasodilation in peripheral resistance vessels has been previously documented [8,9]. Within the extracellular domain of the receptor, *NOTCH3* mutations alter the number of cysteine residues, leading to the abnormal accumulation of the mutated protein in the vascular wall [10]. Misfolding of *NOTCH3* might thus cause an increase in ROS levels also in CADASIL.

The aim of the present study was to verify whether CADASIL patients have increased oxidative stress compared to a control population of unrelated healthy subjects by assessing plasma levels of 3-nytrotyrosine, an index of nitration damage to proteins, and blood and plasma aminothiol concentrations, as markers of oxidant/antioxidant balance.

## Materials and Methods

### Study population

Fifteen subjects, aged 33 to 57 years, with a diagnosis of CADASIL confirmed by the identification of a mutation in the *NOTCH3* gene were included in the study. None of the patients had evidence of autoimmune disorders, liver, or renal diseases. None had experienced a cerebrovascular accident, myocardial infarction or pulmonary embolism within the 3 months preceding the study. None had been taking antioxidant vitamin supplements for at least 2 months.

At enrolment, a detailed clinical history was collected including concomitant treatments and cardiovascular (CV) risk factors, defined according to current guidelines: hypertension (systolic and diastolic blood pressure ≥ 140 and 90 mmHg, respectively or on antihypertensive medications) [11], hyperlipidemia (LDL ≥160 mg/dL or current treatment with lipid-lowering medications) [12], diabetes (documentation of at least two measurements of fasting serum glucose ≥ 126 mg/dL or on antidiabetic drugs) [13], smoking habit. A neurological examination was performed and disability rating scales (Bartel Index and Rankin scale) were computed.

Sixteen unrelated subjects matched by age and gender, with no history, signs or symptoms of cerebrovascular and/or CV disease were enrolled as controls.

The study was approved by the Niguarda Hospital and Istituto Neurologico Carlo Besta Ethics Committee. All subjects signed written consent forms to participate in the study. The investigation conforms to the principles outlined in the Declaration of Helsinki.

### Biochemical assessment

After an overnight fast, an antecubital vein was cannulated and blood was drawn into different pre-chilled Vacutainer tubes for biochemical determinations.

Blood and plasma reduced and total aminothiols (homocysteine, cysteine [Cys], glutathione [GSH], cysteinylglycine) were treated immediately after sample collection and measured by HPLC with fluorescence detection (ProStar, Varian, Surrey, UK) according to methods previously described and validated in our laboratory [14,15].

Plasma 3-nitrotyrosine was measured by using sandwich ELISA kit (Hycult Biotech Inc., Plymouth Meeting, PA) following the manufacturer’s instructions.

Serum glucose, γ-glutamyltransferase (GGT), creatinine, AST, ALT, total cholesterol, HDL-cholesterol, triglycerides, and C reactive protein (CRP) were determined using standard laboratory methods, while vitamin B_12_ and folates were measured by competitive chemiluminescence immunoassay (Roche Diagnostic GmbH, Mannheim, Germany). LDL-cholesterol was calculated using the Friedewald’s method.

### Statistical analysis

Continuous variables are presented as median and interquartile ranges (I–III) or frequency (%). Between-group differences were tested by Mann–Whitney U test for continuous variables, chi-square test or Fisher’s exact test when appropriate for categorical variables. Spearman correlation coefficients were utilized to examine relationships among the aminothiols. Statistical analysis was carried out with the Statistical Package for the Social Sciences (SPSS inc., Chicago, Ill., U.S.) release 17.0 for Windows. The level of significance was set at *P* ≤ 0.05.

## Results

### CADASIL population

Among the 15 enrolled CADASIL patients, a history of migraine was present in 4 subjects (27%), 3 of whom (20%) showed no other clinical manifestation. Previous cerebrovascular events were recorded in 8 (53%) patients: 3 (20%) had had transient ischemic attacks and 5 (33%) a stroke. Four (27%) subjects were asymptomatic mutation carriers. No subject had a Rankin Scale value > 2 and a Barthel Index < 85, in agreement with mild clinical impairment. *NOTCH3* gene mutations were distributed between exon 2 and 19, with the greatest frequency in exon 4 (47%) and were all missense mutations involving a Cys residue.

### Redox balance inCADASIL patients and controls

CV risk factor distribution was similar between CADASIL patients and their gender- and age-matched controls (Table 1). CADASIL patients showed lower systolic blood pressure levels than controls (*P* <0.001). Conversely serum concentrations of total cholesterol, LDL, HDL, triglycerides, fasting glucose, creatinine, AST, ALT, GGT, vitamin B_12_ and folate did not differ.

**Table 1:** Clinical characteristics of study population.

	Controls	CADASIL	p value
	**(*n*=16)**	**(*n*=15)**	
Age, years	44 (39-55)	42 (39-49)	0.580
Male gender, n (%)	8 (50)	10 (67)	0.473
CV risk factors, (any)	11 (69)	6 (40)	0.156
Hypertension, n (%)	5 (31)	2 (13)	0.394
Dislipidemia, n (%)	4 (25)	4 (27)	1.000
Diabetes, n (%)	1 (6)	1 (7)	1.000
Smokers, n (%)	3 (19)	2 (13)	1.000
**Laboratory findings**			
Total cholesterol, (mg/dL)	223 (202-250)	218 (200-243)	0.418
LDL-cholesterol (mg/dL)	140 (117-167)	147 (122-158)	0.800
HDL-cholesterol (mg/dL)	58 (44-73)	49 (42-63)	0.305
Triglycerides (mg/dL)	117 (68-143)	93 (66-134)	0.461
Fasting glucose, (mg/dL)	94 (81-100)	85 (76-94)	0.194
Creatinine (mg/dL)	0.80 (0.65-0.88)	0.89 (0.71-0.99)	0.135
AST (U/L)	20 (17-25)	21 (17-25)	0.861
ALT (U/L)	23 (13-36)	25 (19-31)	0.953
GGT (U/L)	19 (9-32)	23 (13-44)	0.358
Folate (ng/mL)	7.1 (5.3-10.3)	6.6 (4.5-10.2)	0.861
Vitamin B_**12**_, (pg/mL)	447 (358-581)	413 (344-580)	0.813
C-reactive protein (mg/dL)	0.1 (0.0-0.28)	0.1 (0.10-0.13)	0.804
**Vital signs**			
Systolic blood pressure, (mmHg)	132 (125-140)	115 (110-120)	<0.001
Diastolic blood pressure (mmHg)	80 (80-89)	75 (70-85)	0.098
Heart rate (bpm)	66 (60-74)	67 (64-72)	0.771
**Drug therapy**			
ACE inhibitors, n (%)	1 (6)	2 (13)	0.600
Beta-blockers, n (%)	2 (12)	0 (0)	0.484
Calcium antagonists, n (%)	1 (6)	1 (7)	1.000
Statins, n (%)	0 (0)	3 (20)	0.101
Antiplatelets, n (%)	0 (0)	15 (100)	<0.001

Data are presented as number (frequency %) or median (interquartile range); p values are by chi-square test and Mann-Whitney test. CV, cardiovascular; LDL, low-density lipoproteins; HDL, high-density lipoproteins; AST, aspartate amino transferase; ALT, alanine amino transferase; GGT, gamma-glutamyl transferase.

**Figure 1:**
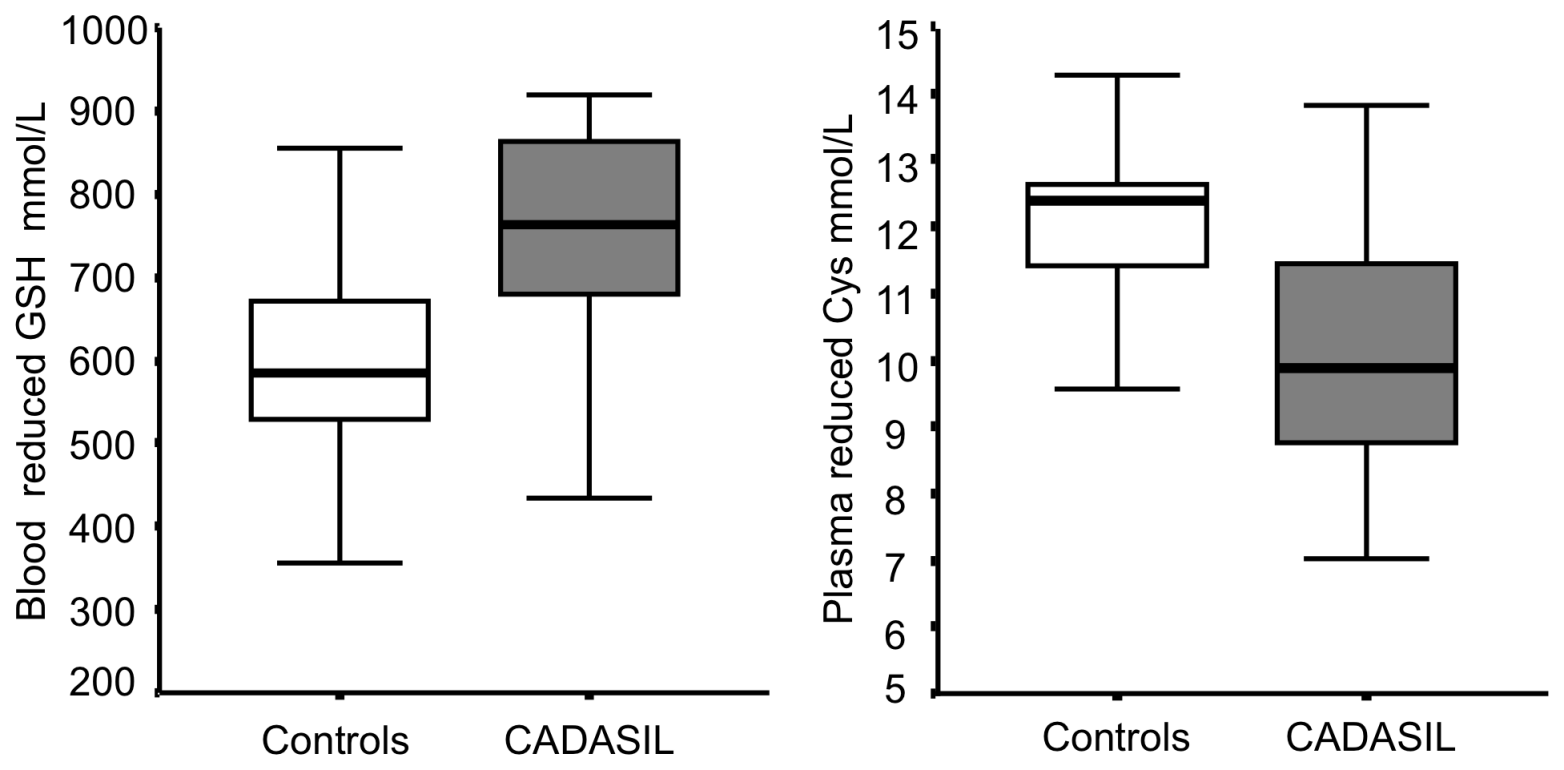
Reduced plasma Cys and blood GSH in controls and CADASIL patients. Box plot of the distribution of reduced plasma Cys and blood GSH concentrations in controls (empty box) and CADASIL patients (dark box). The horizontal line in the middle of each box indicates the median; the top and bottom borders of the box mark the 75^th^ and 25^th^ percentiles, respectively; and the whiskers represent the highest and lowest values that are not outliers or extreme values.

Among markers of oxidative stress (Table 2), only plasma reduced Cys (Pr-Cys) values and blood reduced GSH (Br-GSH) concentrations differed between groups. Patients showed higher Br-GSH levels and lower Pr-Cys compared to controls (Figure 1). No difference were observed in 3-nitrotyrosine values between CADASIL patients and healthy subjects.

**Table 2:** Oxidant/antioxidant balance in controls and in CADASIL patients.

	Controls (*n*=16)	CADASIL (*n*=15)	p value
Pt-Cys (µmol/mL)	244 (208-289)	265 (258-289)	0.406
Pr-Cys (µmol/mL)	12 (11-13)	10 (9-11)	0.010
Pt-CysGly (µmol/mL)	30 (21-37)	33 (28-39)	0.286
Pr Cys–Gly, (µmol/mL)	4.6 (3.3-5.3)	4.3 (2.8-5.1)	0.629
Pt-Hcy (µmol/mL)	8.0 (7.3-8.9)	9.0 (6.7-10.4)	0.423
Pr-Hcy (µmol/mL)	0.18 (0.17-0.20)	0.17 (0.15-0.32)	0.892
Pt-GSH (µmol/mL)	6.3 (4.7-8.6)	6.8 (5.3-7.5)	0.968
Pr-GSH (µmol/mL)	4.0 (1.9-4.9)	3.4 (2.1-5.4)	0.765
Bt-GSH (µmol/mL)	985 (851-1101)	1015 (980-1065)	0.316
Br-GSH (µmol/mL)	584 (527-709)	763 (675-870)	0.019
3-nitrotyrosine, (µmol/mL)	35 (5-66)	28 (16-45)	0.821

Data are presented as median (interquartile range); p values are by Mann-Whitney test. P, plasma; t total; r reduced; B, blood, Cys, cysteine; CysGly, cysteinylglycine; Hcy, homocysteine; GSH, glutathione.

We did not find any correlation between Br-GSH and Pr-Cys in patients (rho 0.25, *P*=0.36), controls (rho -0.21, *P*=0.49), or combined patients and controls (rho -0.15, *P*=0.44).

Within the CADASIL group, no differences in the aminothiol profile and nitrotyrosine levels were found between patients with and those without a history of cerebrovascular events (data not shown).

## Discussion

Since oxidative stress is a prominent abnormality in several neurodegenerative disorders, we investigated the role of this biological mechanism in CADASIL disease. The major novel findings of the present study are that 3-nitrotyrosine, index of oxidative damage to proteins, overlapped between CADASIL and control subjects, whereas, among thiols, lower levels of the oxidant marker Cys and higher concentrations of the antioxidant molecule GSH were found in CADASIL patients than in gender- and age-matched controls. These results point to two different hypothesis: mildly disabled CADASIL patients may exhibit enhanced antioxidant protection or, alternatively, oxidative stress may not represent a peculiar pathophysiologic mechanism in this disease.

Aminothiols are key molecules in redox balance. Cys and homocysteine share a pro-oxidant activity [16], whereas cysteinilglycine and GSH exert antioxidant protection.

**Figure 2:**
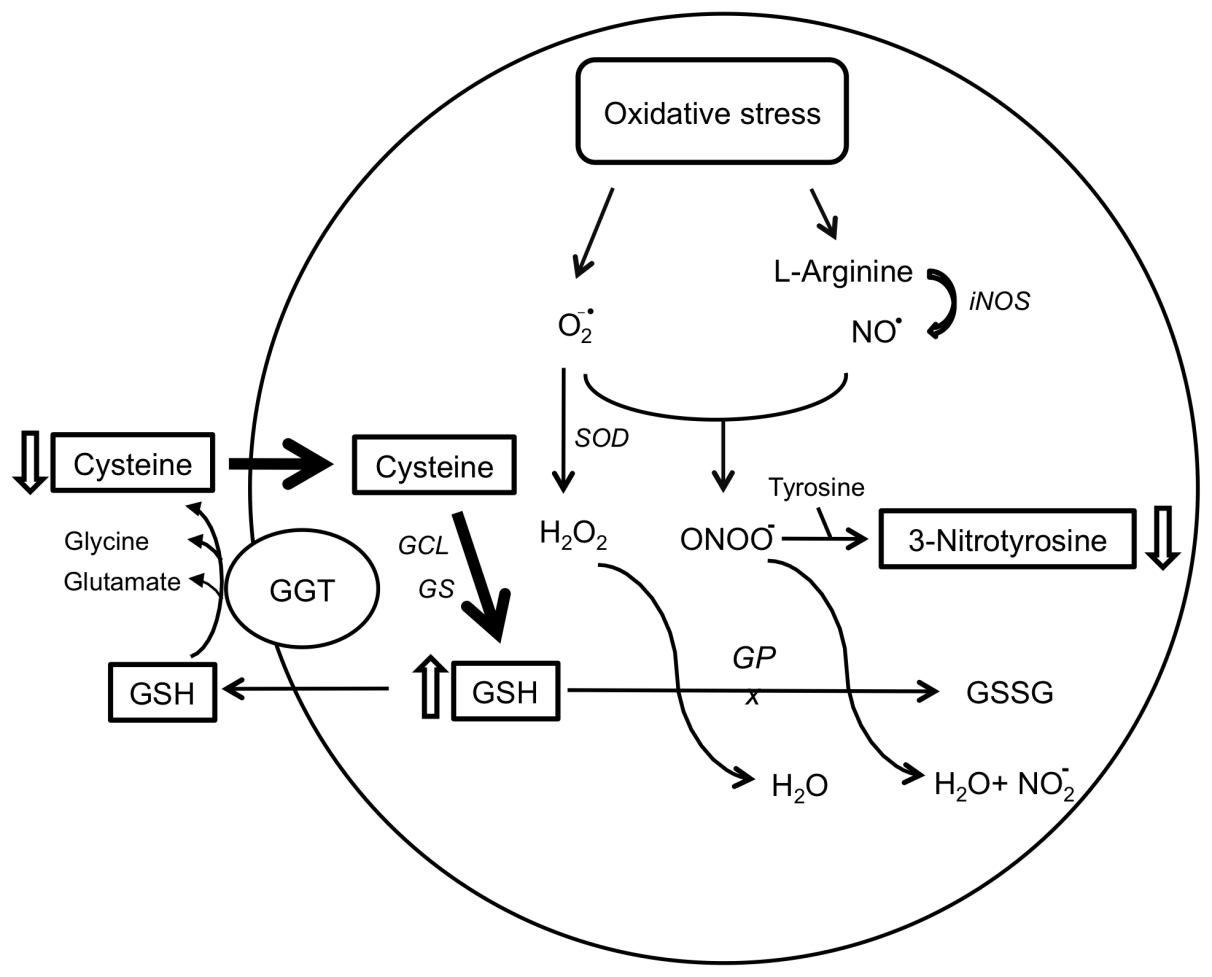
Interplay between oxidative stress and aminothiols. Cysteine is the substrate for the intracellular synthesis of glutathione. Low plasma levels might result from increased influx across the cell membrane and conseuently increased blood glutathione. At the intracellular level glutathione through the enzyme glutathione peroxydase scavenges free radicals such as hydrogen peroxide and peroxynitrite, with a lower production of nitrotyrosine as net effect. CBS, cystathionine-beta-synthase; GCL, glutamate-cysteine ligase; GS, glutathione synthase; GSH, glutathione; GPx, glutathione peroxidase; H_2_O_2_, hydrogen peroxide; iNOS, inducible nitric oxide synthase; MS, methionine synthase; SOD, superoxide dismutase; NO^•^, nitric oxide; NO_2_^-^, nitrite; O_2_^-•^, superoxide anion.

The potential vascular toxicity of Cys has been emphasized in several works. In vitro, Cys exhibits auto-oxidation properties in the presence of metal ions, with the ensuing generation of free radicals species [16]. Cys was shown to produce, by inhibiting the basally released endothelium-derived relaxing factor, a dose-dependent contraction increase in the endothelium [17]. Previous studies have reported increased plasma total Cys levels in patients with myocardial infarction, cerebral infarction or peripheral vascular disease [18]. However, the reduced form of Cys is also one of the substrates for the synthesis of GSH [19], which is produced from the precursors gamma-glutamate, cysteine and glycine through the enzymatic action of both glutamate-cysteine ligase and GSH synthase. The low Pr-Cys concentrations found in our CADASIL patients could thus be the result of an increased metabolic rate of intracellular GSH production (Figure 2): Br-GSH concentrations were in fact higher in CADASIL compared to healthy subjects in our study. Since the Cys pool substrate for Br-GSH synthesis includes both extracellular (Pr-Cys) and intracellular Cys, the lack of correlation between Br-GSH and Pr-Cys observed in both patients and controls does not allow to rule out this hypothesis.

GSH has several major physiological functions: it maintains SH groups of proteins in a reduced state, it participates in amino acid transport, it detoxifies foreign compounds and enzymatically degenerates endogenous peroxides, it forms bioactive molecules and acts as a coenzyme in several enzymatic reactions [20]. GSH is a free radical scavenger and a proton donor for GSH peroxidase that is known to play a neuroprotective role [21]. Reduced GSH levels have been found in a number of neurodegenerative diseases that present oxidative stress, as well as in the process of normal aging [22].

Changes of GSH metabolism have been observed in moderate and severe ischemic stroke [23]. Increased activities of GSH peroxidase and GSH transferase are the most typical changes, but an increase in GSH reductase and GSH was also observed [24]. Ozkul et al. reported high GSH concentrations with concomitantly high malondialdehyde levels in patients with acute ischemic stroke [25].

The GSH system has been supposed to play an important role in brain tolerance to ischemia. The high intracellular GSH levels found in our patients might represent a defensive mechanism against the chronic cerebral hypoperfusion that characterizes CADASIL disease. Cerebrovascular dysfunction in addition to microcirculatory rarefaction are, in fact, the earliest consequences of pathogenic mutant *NOTCH3* expression [7].

In contrast with other evidences, we did not find any increase in pro-oxidant aminothiols and nitrotyrosine as oxidative stress marker in CADASIL patients. Ragno et al. observed changes in telomere length of peripheral blood leukocytes, a condition that is often associated with high levels of oxidative stress and chronic inflammation [26]. Ferrer et al. reported a link between vascular pathology and semicarbazide-sensitive amine oxidase (SSAO) overexpression [27]. This enzyme, which is selectively expressed in human brain blood vessels, but not in other cellular components (e.g., neurons and glial cells), plays a role as mediator in reactions involving H_2_O_2_ production [28]. Moreover, Formichi et al. have reported an increase of oxidative stress-induced apoptosis in peripheral blood leukocytes and fibroblasts from CADASIL patients [29].

Conversely, our CADASIL patients and healthy controls had similar levels of plasma 3-nitrotyrosine, a sensitive marker of oxidative stress. This compound results from the oxidative interaction between nitric oxide and superoxide anion, that produces the highly reactive peroxynitrite, a potent oxidizing agent. Peroxynitrite initiates lipid peroxidation in biological membranes, hydroxylation and nitration of aromatic amino acid residues, and sulfhydryl oxidation of proteins [30]. Although 3-nitrotyrosine has been confirmed to be a sensitive indicator of peroxynitrite generation in ischemic stroke and in neurodegenerative disorders [31], the analysis of other markers might instead reveal oxidative imbalance in CADASIL patients.

Since *NOTCH3* mutations cause ubiquitous degeneration of vascular smooth muscle cells [7], we studied oxidative stress markers at the systemic rather than cerebral level. Previous studies in neurodegenerative and cerebrovascular disorders found evidence of increased oxidative stress changes in peripheral blood samples [2,3]. Negative findings from the peripheral circulation in our population cannot definitely rule out the possibility that local vascular redox imbalance may indeed be present in the small vessels of the brain, but not be reflected by blood level changes. Increased oxidative stress, as expressed by immunoistochemical SSAO determination, has been proven in autoptic brain tissue samples of CADASIL patients, who died at an advanced stage of their disease, comparable to patients with Alzeihmer’s disease [27]. On the other hand, systemic vascular abnormalities have been consistently observed in CADASIL patients. Structural small vessels changes have been documented in skin biopsy samples [7] and endothelial function has been previously shown to be altered in small peripheral resistance vessels in a substantial proportion of CADASIL patients [8,9] and to correlate with reduced intracranial vasoreactivity [32]. This notion suggests that vascular abnormalities could indeed be reflected by systemic biochemical alterations.

ROS are key signalling molecules in neurohumoral mechanisms involved in blood pressure regulation, volume homeostasis, baroreflex function and sympathetic activity [33,34]. Inhibition of ROS-producing enzymes, antioxidants and ROS scavengers lower blood pressure whereas pro-oxidants increase, and free radicals have been causally associated with hypertension in animal models. Oxidative stress in the central nervous system is implicated in the neuro-dysregulation associated with some forms of hypertension. Our CADASIL patients showed instead significantly lower blood pressure values than controls, consistently with previous reports [35]. Relative hypotension in these patients is postulated to derive from functional failure of brain structures and connections controlling circadian blood pressure variations, secondary to white matter damage. Conversely, at the relatively early stage of clinical presentation in our series, it is also possible that the increased antioxidant response might be a contributory mechanism to hypotension, since at low concentrations oxidants act as signaling molecules for vascular tone regulation [1].

Some limitations of our study should be considered.

The sample was relatively small, as it commonly occurs in similar studies on patients affected by such a rare disease. An imbalance in drug treatment was also observed: 80% of CADASIL patients and none of controls were taking aspirin and 20% statins, drugs which are known to have antioxidant properties [36,37] and might have damped deleterious oxidative stress signals.

We did not measure the activity of enzymes involved in GSH metabolism. However we evaluated the direct product of their action, the intracellular GSH concentration, so we discuss the net effect of possible changes in enzyme activities.

In conclusion, the high levels of antioxidant molecules and low or similar levels of oxidant mediators found in our CADASIL population as compared with control subjects point to two possible hypotheses. These findings might be the expression of an effective protective action against free radical formation at an early stage of clinical symptoms. Alternatively, our results may also support the hypothesis that oxidative stress is not directly involved in the pathogenesis of CADASIL and that antioxidant changes may be related to other biological mechanisms.

Further evaluation of multiple oxidative stress markers and endogenous antioxidant capacity in a larger population of CADASIL patients is warranted. 
